# Pyomelanin from *Pseudoalteromonas lipolytica* reduces biofouling

**DOI:** 10.1111/1751-7915.12773

**Published:** 2017-08-22

**Authors:** Zhenshun Zeng, Xing‐Pan Guo, Xingsheng Cai, Pengxia Wang, Baiyuan Li, Jin‐Long Yang, Xiaoxue Wang

**Affiliations:** ^1^ Key Laboratory of Tropical Marine Bio‐resources and Ecology Guangdong Key Laboratory of Marine Materia Medica RNAM Center for Marine Microbiology South China Sea Institute of Oceanology Chinese Academy of Sciences Guangzhou China; ^2^ Key Laboratory of Exploration and Utilization of Aquatic Genetic Resources International Research Center for Marine Biosciences Shanghai Ocean University Shanghai China

## Abstract

Members of the marine bacterial genus *Pseudoalteromonas* are efficient producers of antifouling agents that exert inhibitory effects on the settlement of invertebrate larvae. The production of pigmented secondary metabolites by *Pseudoalteromonas* has been suggested to play a role in surface colonization. However, the physiological characteristics of the pigments produced by *Pseudoalteromonas* remain largely unknown. In this study, we identified and characterized a genetic variant that hyperproduces a dark‐brown pigment and was generated during *Pseudoalteromonas lipolytica* biofilm formation. Through whole‐genome resequencing combined with targeted gene deletion and complementation, we found that a point mutation within the *hmgA* gene, which encodes homogentisate 1,2‐dioxygenase, is solely responsible for the overproduction of the dark‐brown pigment pyomelanin. In *P. lipolytica*, inactivation of the *hmgA* gene led to the formation of extracellular pyomelanin and greatly reduced larval settlement and metamorphosis of the mussel *Mytilus coruscus*. Additionally, the extracted pyomelanin from the *hmgA* deletion mutant and the *in vitro*‐synthesized pyomelanin also reduced larval settlement and metamorphosis of *M. coruscus*, suggesting that extracellular pyomelanin released from marine *Pseudoalteromonas* biofilm can inhibit the settlement of fouling organisms.

## Introduction

Marine biofouling communities are surface‐dwelling communities composed of all types of marine bacteria, invertebrates and diatoms, and interactions among these organisms govern the nature of biofouling communities (Mieszkin *et al*., 2013; Lee *et al*., 2014; Dang and Lovell, 2016). Microfouling organisms located on the surface of a marine substratum create serious issues worldwide, including an increase in drag force and metal corrosion, as well as a reduction in the efficiency of energy transfer (Dobretsov *et al*., 2013; Karpov *et al*., 2014b). Tributyltin‐based antifouling technologies have been considered the most effective approach for preventing the accumulation of fouling organisms (Omae, 2003; Chambers *et al*., 2006). In September 2008, the use of organotin compounds was banned due to their adverse effects on non‐target marine organisms and ecological environments. The discovery of natural antifouling compounds has received growing attention due to the global efforts being made to develop more environmentally friendly antifouling technologies (Yebra *et al*., 2004; Qian *et al*., 2010, 2015).

*Pseudoalteromonas* is a genus of Gammaproteobacteria that is commonly found in various marine environments (Gauthier *et al*., 1995; Wietz *et al*., 2010; Salazar *et al*., 2016). *Pseudoalteromonas* spp. possess important features that have drawn increasing interest from researchers in the ecological and pharmaceutical sciences: (i) they are often found in association with eukaryotic hosts, including marine phytoplankton, sponges, mussels, scallops, tunicates and corals (Holmstrom and Kjelleberg, 1999); (ii) they produce a variety of biologically active extracellular compounds, including pigmented secondary metabolites, extracellular enzymes and extracellular polysaccharide (EPS) (Kalinovskaya *et al*., 2004; Bowman, 2007). The colonization of marine surfaces by marine bacteria often involves biofilm formation, which aids survival in competitive environments or serves as a cue to settlement by invertebrate larvae (Qian *et al*., 2007; Egan *et al*., 2008). Biofilms formed by *Pseudoalteromonas* produce bioactive compounds that inhibit the settling of various fouling invertebrates and algae (Holmstrom and Kjelleberg, 1999; Egan *et al*., 2002; Bowman, 2007). We recently reported the occurrence of morphological diversification in EPS production during the biofilm formation of various *Pseudoalteromonas* spp. (Zeng *et al*., 2015). In particular, we found that genetic variants generated in *Pseudoalteromonas lipolytica* biofilms with alteration in capsular polysaccharide or cellulose production exhibit strong antifouling activities, which indicates that biofilm variants might produce extracellular compounds with enhanced antifouling activities (Zeng *et al*., 2015).

The genus of *Pseudoalteromonas* has been divided into pigmented and non‐pigmented clades (Bowman, 2007). A correlation between the antifouling activities of *Pseudoalteromonas* and pigment expression has been observed by several research groups (Egan *et al*., 2002; Bowman, 2007; Huang *et al*., 2011). However, the molecular mechanisms underlying the secretion of pigmented secondary metabolites by *Pseudoalteromonas* and the regulation of pigment production remain largely unknown. In this study, we isolated a pigmented variant during *P. lipolytica* biofilm formation and found that the release of dark‐brown pigments was caused by a mutation in the *hmgA* gene, which encodes homogentisate 1,2‐dioxygenase and that this mutation led to the hyperproduction of pyomelanin. Deletion of the *hmgA* gene in *P. lipolytica* reduced the larval settlement and metamorphosis of the mussel *Mytilus coruscus*, a significant biofouling organism found in China, and this finding suggests that extracellular pyomelanin released from marine bacteria can inhibit the settlement of fouling organisms.

## Results

### Pigmentation during *P. lipolytica* biofilm formation

**Figure 1:**
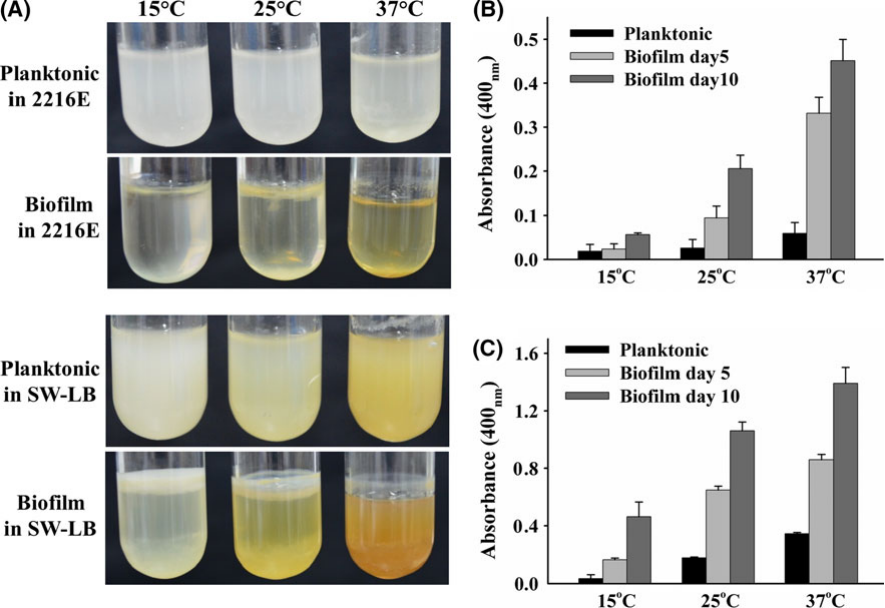
Pigmentation occurring during *P. lipolytica* biofilm formation. A. A substantial amount of pigments are produced during biofilm formation for 10 days at different growth temperatures. B. Quantification of pigment production in 2216E medium. C. Quantification of pigment production in SW‐LB medium. Data are from three independent cultures. One SD is shown in B and C, and representative images are shown in A.

Like other marine Gammaproteobacterial strains, *P. lipolytica* is able to form pellicle‐type biofilm due to the establishment of oxygen and nutrient gradients at the interface of the air in the nutrient‐rich medium SW‐LB (sea water LB) or nutrient‐poor medium 2216E marine broth (Armitano *et al*., 2014; Zeng *et al*., 2015). Two types of genetic variants with relatively high proportions were recently characterized during *P. lipolytica* biofilm formation, and the results demonstrated that each type of variant leads to changes in the colony morphology from smooth to wrinkled and opaque to translucent (Zeng *et al*., 2015). In addition to these changes in the colony morphology of *P. lipolytica*, we observed an increased amount of brownish pigments of a later‐stage biofilm compared with that of planktonic cultures (Fig. 1A). We then quantified the level of brownish pigment in the supernatant by measuring the absorbance at 400 nm. When cultured in 2216E medium, *P. lipolytica* produced a eightfold higher amount of this brownish pigment after prolonged static incubation (day 10) at 25 °C (Fig. 1B), and similar trends were also observed when cultured in nutrient‐rich SW‐LB medium (Fig. 1C). In addition, the release of this brownish pigment was further enhanced by culturing at a higher temperature during biofilm formation (Fig. 1). Compared with that observed at 15 °C, the brownish pigment production was increased 39‐fold after prolonged static incubation (day 10) at 37 °C in SW‐LB medium (Fig. 1C), suggesting that the production of brownish pigment by *P. lipolytica* is induced during biofilm formation and in response to the upshift of temperature.

### Pigmented variants isolated from *P. lipolytica* biofilms

**Figure 2:**
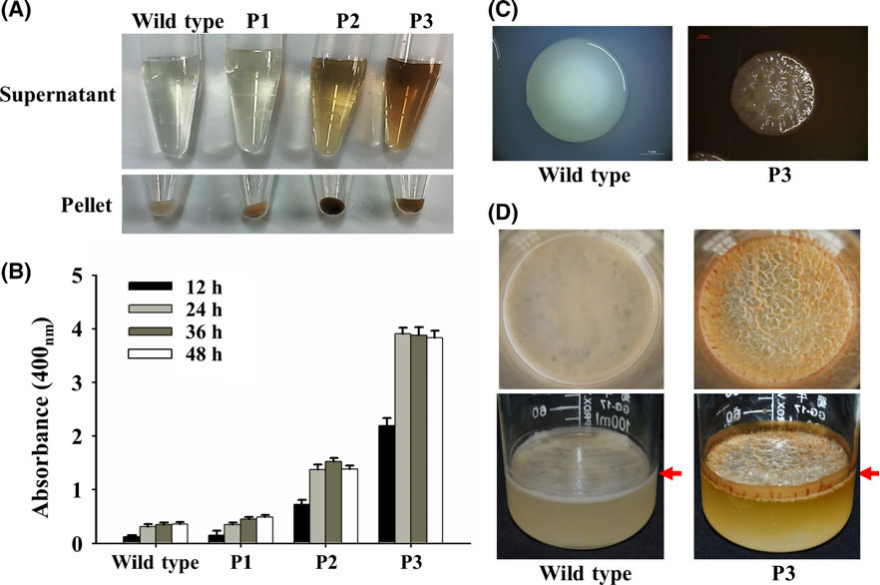
Three pigment production variants isolated from a biofilm population. A. Supernatant and the cells pellet collected from the cultures of the wild‐type and pigmented strains, which were isolated from a biofilm population. B. Quantification of pigment production by the three variants cultured in SW‐LB medium. C. Colony morphology of wild‐type and P3 variant cells cultured on SW‐LB agar plates. D. Pellicle formation of wild‐type and P3 variant cells in SW‐LB medium is marked with a red arrow bar. Data are from three independent cultures. One SD is shown in B, and representative images are shown in A, C and D.

Genetic variants generated during biofilm formation have been shown to account for the phenotypic changes observed in biofilm cells compared with planktonic cells (Stewart and Franklin, 2008; Zeng *et al*., 2015). Thus, we aimed to determine whether pigmented variants are also generated during *P. lipolytica* biofilm formation. A single colony of wild‐type *P. lipolytica* was propagated in SW‐LB medium at 25 °C for 10 days without shaking to allow formation of a biofilm. The biofilm cells were then destructively sampled, and randomly selected cells were plated onto SW‐LB agar for morphological examination. As reported earlier, the majority of phenotypic variants showed a wrinkled or translucent morphology compared with the wild‐type ancestral cells, which presented a smooth and opaque morphology (Zeng *et al*., 2015). Of the 5000 randomly selected cells, three cells presented a change from a non‐pigmented morphology to brownish pigment morphology (Fig. 2A). These pigmented variants (named as P1, P2 and P3) were re‐inoculated into fresh medium for three rounds of overnight passaging, and the production of brownish pigment remained constant, suggesting that genetic changes had occurred. We then quantified the production of brownish pigment by these variants by measuring the absorbance of the supernatants of the liquid cultures after prolonged incubation at 25 °C. Although the P1 variant appeared brownish on the agar plate, it did not show increased pigment production in the supernatant of the liquid culture compared with the wild‐type strain, indicating that P1 mostly produces intracellular pigments (Fig. 2A). In contrast, the P2 and P3 variants showed significantly higher production in the supernatants after shaking culture compared with the wild‐type strain, and the P3 variant produced a 2.8 ± 0.7‐fold higher amount of pigment than the P2 variant (Fig. 2B). Additionally, an extended incubation of the P3 variant resulted in a visible release of the brownish pigment into solid agar, but this visible release was not observed with the wild‐type strain; however, the colours of the colonies of the two strains were comparable, suggesting that the P3 variant mostly produces extracellular pigments (Fig. 2C). When cultured statically in liquid culture, the P3 variant showed the formation of a more brownish and thicker pellicle along with an increased release of brownish pigment into the supernatant (Fig. 2D). Collectively, these results demonstrate that genetic variants that exhibit hyperproduction of this brownish pigment are naturally generated during the formation of *P. lipolytica* biofilms.

### Point mutation in *hmgA* leads to pyomelanin overproduction

**Figure 3:**
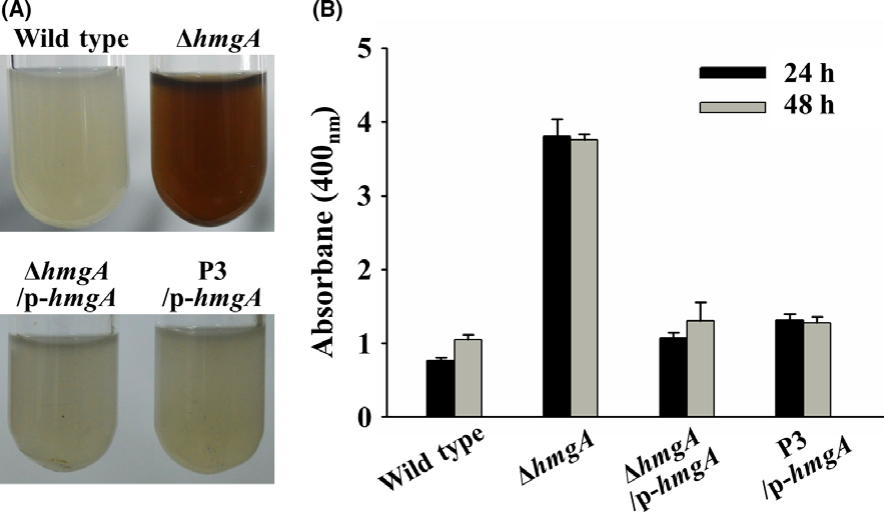
A mutation in *hmgA* leads to pigment production. A. The deletion of *hmgA* in *P. lipolytica* produces the brownish pigment. Complementation of *hmgA* restores the phenotypes of the Δ*hmgA* mutant and the P3 variant to that of the non‐pigmented wild type. B. Quantification of pigment production in SW‐LB medium. Data are from three independent cultures. One SD is shown in B, and representative images are shown in A.

We subsequently explored the genomic changes that had occurred in the genome of the P3 variant through whole‐genome resequencing. The P3 variant was resequenced using the Illumina HiSeq 2000 platform, and 454 Mb of sequencing data was produced. In general, the depth of the whole‐genome resequencing exceeded 98%, and the coverage exceeded 99.82%, suggesting that the sequence data were of high quality. In summary, 12 point mutations (designated M1 to M12) were identified in the P3 strain, and these included eight non‐synonymous mutations and one nonsense mutation (Table S1). Among the non‐synonymous mutations, we found that M11 was located in the coding region of the *hmgA* gene, which encodes homogentisate 1,2‐dioxygenase (HmgA). HmgA catalyses the conversion of homogentisic acid (HGA) into 4‐maleylacetoacetate (Arias‐Barrau *et al*., 2004; Wang *et al*., 2015a,b). The accumulation of HGA can lead to the formation of pyomelanin due to autopolymerization in closely related marine bacteria, such as *Vibrio, Pseudomonas* and *Shewanella* (Coon *et al*., 1994; Wang *et al*., 2013; Hocquet *et al*., 2016). Thus, we reasoned that the brownish pigment produced by the P3 variant could be due to the inactivation of HmgA. To test this hypothesis, we first constructed an in‐frame deletion of the *hmgA* gene in *P. lipolytica* (Wang *et al*., 2015a,b). As expected, the deletion mutant Δ*hmgA* produced a significantly higher amount of extracellular brownish pigment than that of the wild‐type strain when grown planktonically (Fig. 3). Expression of *hmgA in trans* via the pBBR1MCS*‐hmgA* plasmid under the control of its own promoter restored the morphology of the Δ*hmgA* mutant and the P3 variant to that of the non‐pigmented wild type (Fig. 3), suggesting that the brownish pigment produced by the P3 variant is due to the inactivation of HmgA. Moreover, the pigment produced by the Δ*hmgA* mutant exhibited the highest absorbance at 400 nm in visible light spectrum, which was the same as the pigments generated by the biofilm (Fig. S1), suggesting that the brownish pigment should contain same or similar compounds (Ruzafa *et al*., 1995; Lagunas‐Munoz *et al*., 2006; Chatfield and Cianciotto, 2007).

To further confirm the brownish pigment was pyomelanin, pigment produced by Δ*hmgA* mutant and the synthetic HGA‐based pyomelanin were extracted and analysed by Fourier transform infrared spectrometer (FTIR) spectroscopy which is regarded as the most informative method for the structural analysis of melanins (Bilinska, 1996; Schmaler‐Ripcke *et al*., 2009). The dried extracted pigment resulted in a black powder with the following solubility properties indicative of melanin: insoluble in organic solvents (ethanol, chloroform and acetone) and soluble in NaOH solutions with a pH of 10 (data not shown) (Turick *et al*., 2002). The FTIR spectra of the brownish pigment extracted from the *P. lipolytica* Δ*hmgA* mutant shared definite signature peaks as previously reported for *Shewanella algae* pyomelanin and synthetic pyomelanin produced by auto‐oxidation of HGA (Turick *et al*., 2002) (Fig. S2). The peaks at the following wave numbers and their corresponding structures include the bands at 3300 cm^−1^, 2900 cm^−1^ and 1560 cm^−1^. Moreover, the fingerprint regions between 500 cm^−1^ and 1000 cm^−1^ of these samples resemble each other (Turick *et al*., 2002; Schmaler‐Ripcke *et al*., 2009) (Fig. S2). Collectively, these results demonstrate that the pigment produced by Δ*hmgA* mutant shared key characteristics of known melanin and also shared a high degree of similarity to the synthesized pyomelanin.

### l‐tyrosine metabolism is involved with production of pyomelanin

**Figure 4:**
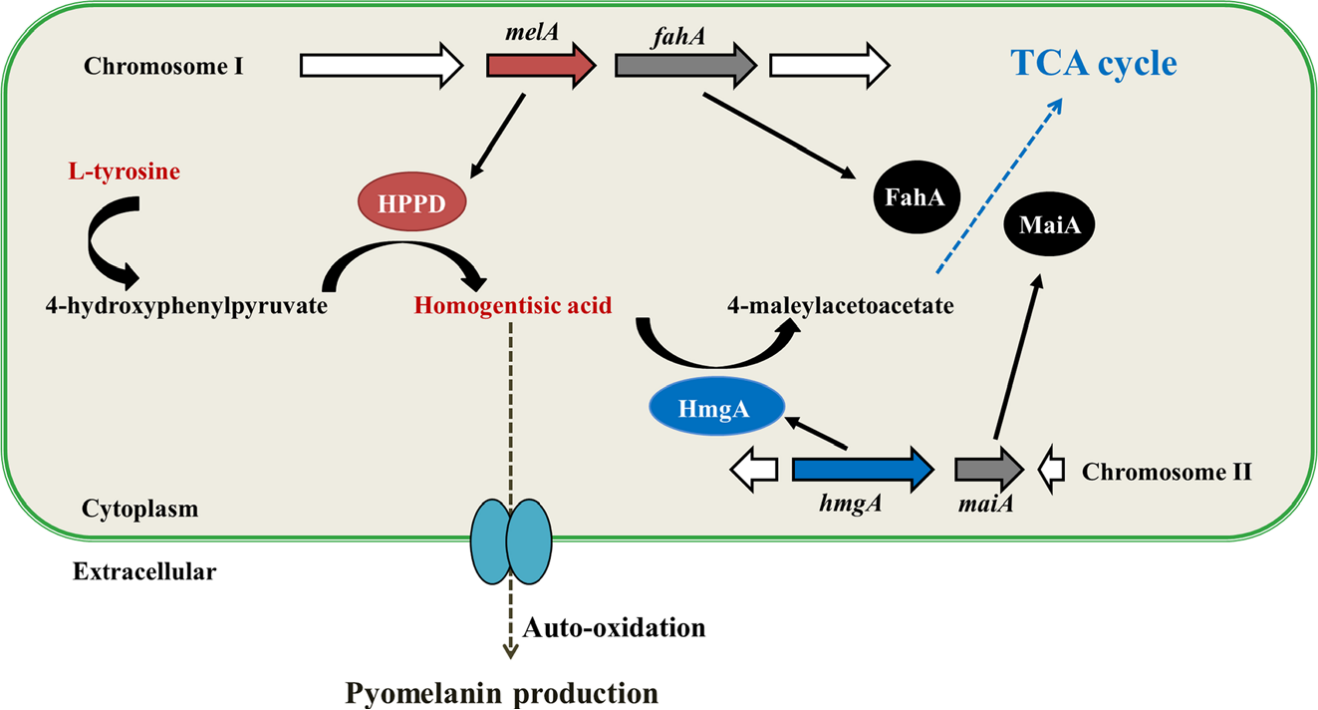
Metabolic pathway for pyomelanin production in *Pseudoalteromonas*. Four genes participating in the l‐tyrosine catabolic pathway were found in the *Pseudoalteromonas* genome: two of these genes, *melA* and *fahA*, are located on chromosome I, and the other two, *hmgA* and *maiA*, are located on chromosome II. Inactivation of the *hmgA* gene could lead to accumulation of the intermediate homogentisic acid, and auto‐oxidization of homogentisic acid would lead to the production of extracellular pyomelanin.

**Figure 5:**
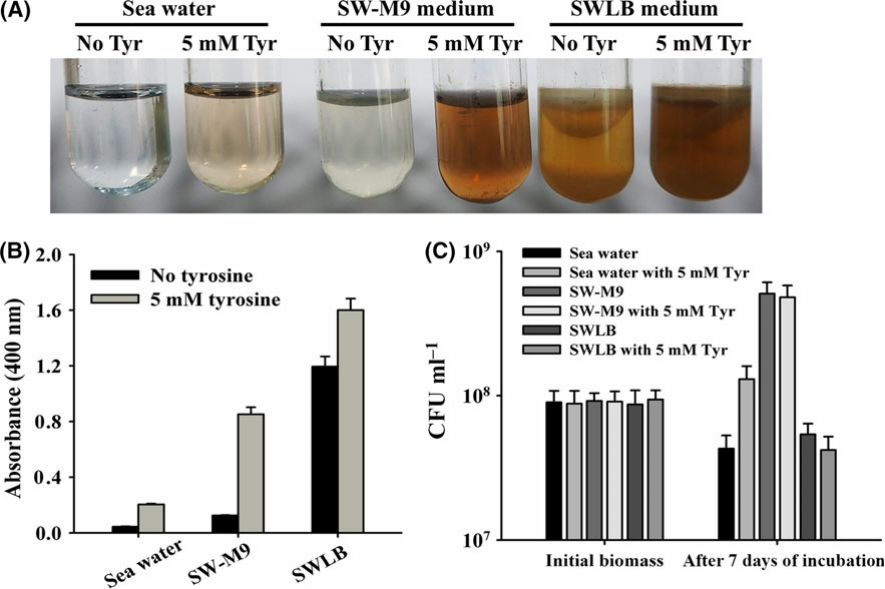
l‐tyrosine metabolism contributes to pigmentation during biofilm formation. A. Pigment production by the wild‐type strain after static incubation in sea water, SW‐M9 and SW‐LB medium with and without l‐tyrosine for 7 days at 37 °C. B. Quantification of pigment production by the wild‐type strain after static incubation with and without l‐tyrosine. C. The cell density (CFU ml^−1^) at initial inoculation and after incubation for 7 days. Data are from three independent cultures. One SD is shown in B and C, and representative images are shown in A.

HmgA is an important enzyme that participates in the l‐tyrosine catabolic pathway (Arias‐Barrau *et al*., 2004). Four genes participating in the l‐tyrosine catabolic pathway are present in *P. lipolytica* (Table S2) and other *Pseudoalteromonas* species that have been isolated from diverse marine environments, including extreme environments, such as SM9913, suggesting that this pathway is conserved in *Pseudoalteromonas* (Table S3). The distribution of the four enzymes belonging to the l‐tyrosine catabolism pathway on two different chromosomes is also a common feature in these *Pseudoalteromonas* strains. The *melA* gene (*AT00_02290* on chromosome I) encodes 4‐hydroxyphenylpyruvate dioxygenase (4‐HPPD), which is responsible for the synthesis of HGA. The *hmgA* gene (*AT00_15690* on chromosome II) is responsible for the degradation of HGA to 4‐maleylacetoacetate. The two other genes, *fahA* (*AT00_02285* on chromosome I) and *maiA* (*AT00_15695* on chromosome II), encode enzymes that convert 4‐maleylacetoacetate to acetoacetate, and fumarate substrates that are subsequently utilized in the tricarboxylic acid (TCA) cycle (Fig. 4) (Schmaler‐Ripcke *et al*., 2009). We observed that the pyomelanin production in the biofilm of *P. lypolytica* was increased under elevated temperature (Fig. 1). Thus, we measured the expression of two key genes involved in the production of HGA under higher temperature (37 °C) and found that the production of the pyomelanin in *P. lypolytica* is neither due to a reduced expression of *hmgA* gene which is responsible for HGA degradation nor due to an increased expression of the *melA* gene which is responsible for the HGA synthesis (Table S5). Thus, we reasoned that the increased production of pigments in biofilms and under high temperature might involve with the generation of the genetic variants with increased pigment production in *P. lypolytica*. To further test whether pyomelanin is produced through the l‐tyrosine catabolic pathway in *P. lipolytica*, we tested the pyomelanin production of the *P. lipolytica* by culturing statically through the addition of 5 mM l‐tyrosine. In sea water where no l‐tyrosine is present, the addition of l‐tyrosine not only induced pyomelanin production (Fig. 5A and B) but also promoted cell growth (Fig. 5C). In SW‐M9 medium where 0.2% casamino acid was added into sea water or in SW‐LB medium, the addition of l‐tyrosine induced pyomelanin production (Fig. 5A and B) without affecting cell growth (Fig. 5C). These results suggest that the production of pyomelanin is induced by the presence of more l‐tyrosine.

### Deletion of *hmgA* reduces plantigrade settlement of *M. coruscus*

**Figure 6:**
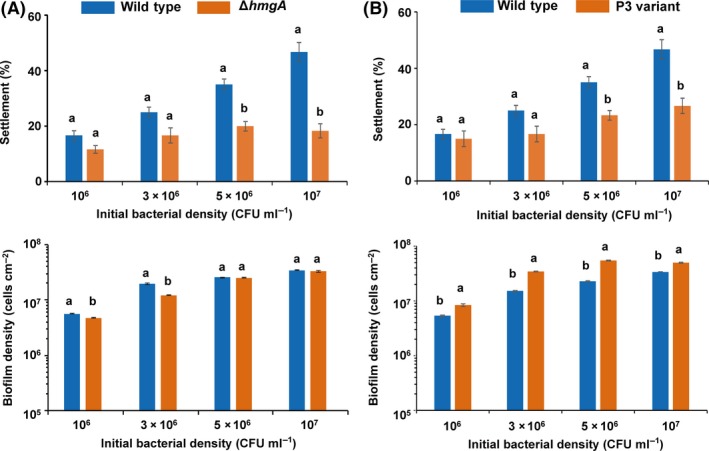
Both of the *hmgA* deletion strain and P3 variant reduce plantigrade settlement of *M. coruscus*. A. Plantigrade settlement‐inducing activities of the attached biofilms formed by the wild‐type and Δ*hmgA* strains with different initial CFUs (upper panel). Cell densities of the attached biofilms formed by the wild‐type and Δ*hmgA* strains on glass slips with different initial CFUs (lower panel). B. Plantigrade settlement‐inducing activities of the attached biofilms formed by the wild‐type and the P3 variant strains with different initial CFUs (upper panel). Cell densities of the attached biofilms formed by the wild‐type and the P3 variant strains on glass slips with different initial CFUs (lower panel). The lowercase letters a and b indicate a significance level of *P* < 0.05.

Because the antifouling activities of *Pseudoalteromonas* are often associated with pigmentation, we then explored whether pyomelanin overproduction is associated with the antifouling activities of *P. lipolytica*. Hence, we first tested whether biofilms formed by the Δ*hmgA* mutant affect the plantigrade settlement of *M. coruscus*. Biofilms of the wild‐type and Δ*hmgA* strains were prepared using the same concentration of bacterial suspensions, and attached biofilms that formed on glass slips after 2 days of incubation were used for the settlement assay. The results showed that at initial concentrations of 5 × 10^6^ CFU ml^−1^ and 1 × 10^7^ CFU ml^−1^, the plantigrade settlement‐inducing activities of the attached biofilms formed by Δ*hmgA* were reduced 42% and 62% compared with that of the wild‐type strain respectively (Fig. 6A, upper panel, Kruskal–Wallis test, *P* < 0.001). Moreover, no significant difference in biofilm density was observed between the Δ*hmgA* mutant and the wild‐type strain at the initial concentrations of 5 × 10^6^ CFU ml^−1^ and 1 × 10^7^ CFU ml^−1^ respectively (Fig. 6A, lower panel, Kruskal–Wallis test, *P* > 0.05). In addition, we also tested the antifouling activity of the P3 variant. Similar results in terms of plantigrade settlement were found for the comparison of the P3 variant with the wild‐type strain: the inducing activities of the P3 variant were reduced 34% and 42% at the initial concentrations of 5 × 10^6^ CFU ml^−1^ and 1 × 10^7 ^CFU ml^−1^ respectively (Fig. 6B, upper panel, Kruskal–Wallis test, *P* < 0.00). We also quantified the cell densities of the attached biofilms from the two strains, and the results showed that the density of attached cells in the biofilm of the P3 variant was slightly higher than that found for the wild type (Fig. 6B, lower panel, Kruskal–Wallis test, *P* < 0.001), suggesting that the decreased plantigrade settlement percentage obtained with the P3 variant was not due to a decrease in the cell density of the attached biofilm. Collectively, these results indicate that pyomelanin production leads to increased activities against *M. coruscus* settlement.

### Deletion of *hmgA* reduces larval settlement and metamorphosis of *M. coruscus*

**Figure 7:**
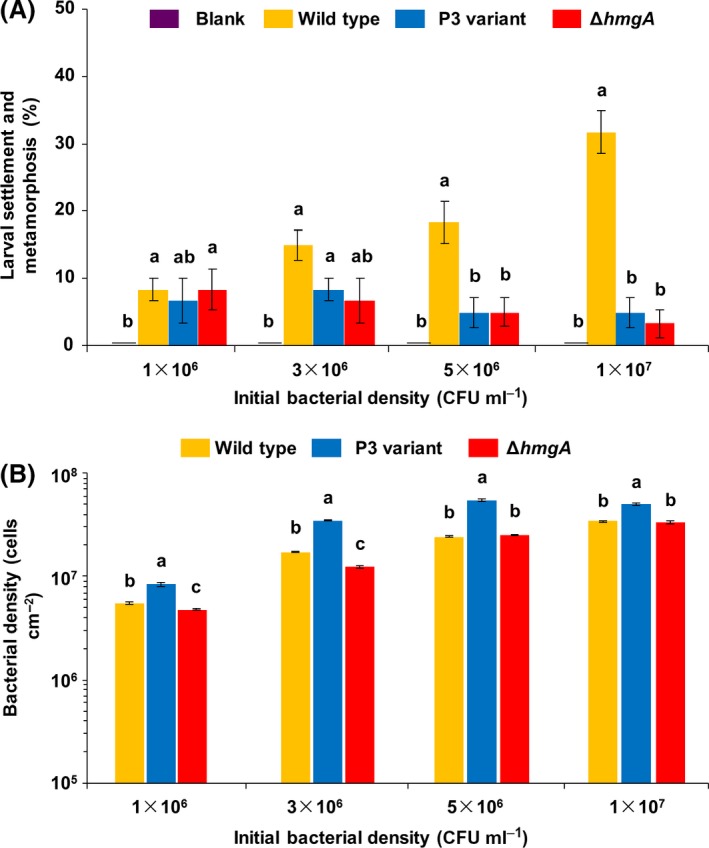
Both of the *hmgA* deletion strain and P3 variant reduce larval settlement and metamorphosis of *M. coruscus*. A. Larval settlement‐inducing activities of the attached biofilms formed by the wild‐type, P3 variant (pyomelanin hyperproduction variant) and Δ*hmgA* mutant strains (upper panel) with different initial CFU respectively. B. Cell densities of the attached biofilms formed by the wild‐type, P3 variant and Δ*hmgA* mutant strains on surfaces of 24‐well plates with different initial CFU. The lowercase letters indicate a significance level of *P* < 0.05.

We next tested whether biofilms formed by the Δ*hmgA* mutant and the pyomelanin hyperproduction variant impact larval settlement and metamorphosis of *M. coruscus*. In the case of initial concentrations of 5 × 10^6^ CFU ml^−1^, the percentages of larval settlement and metamorphosis in biofilms developed by Δ*hmgA* and pyomelanin hyperproduction variant were reduced 73% and 73% compared with that of the wild‐type strain respectively (Fig. 7A, Kruskal–Wallis test, *P* < 0.001). In the case of the initial concentrations of 1 × 10^7^ CFU ml^−1^, the percentages of larval settlement and metamorphosis in biofilms developed by pyomelanin hyperproduction variant and Δ*hmgA* were reduced 84% and 90% compared with that of the wild‐type strain respectively (Fig. 7A, Kruskal–Wallis test, *P* < 0.001). Moreover, higher biofilm density in the pyomelanin hyperproduction variant and lower biofilm density in the Δ*hmgA* mutant were observed when compared with that of the wild‐type strain at the initial concentrations of 1 × 10^6^ CFU ml^−1^ (Fig. 7B, Kruskal–Wallis test, *P* < 0.05). In the case of the initial concentrations of 5 × 10^6^ CFU ml^−1^ and 1 × 10^7 ^CFU ml^−1^, higher biofilm density in the pyomelanin hyperproduction variant was observed (Kruskal–Wallis test, *P* < 0.05), while no significant difference was observed between the Δ*hmgA* mutant and the wild‐type strain (Kruskal–Wallis test, *P* > 0.05). It suggests that the decreased larval settlement and metamorphosis was not due to a change in the cell density of the attached biofilm. Collectively, these results indicate that pyomelanin production leads to increased activities against *M. coruscus* settlement.

### Inhibiting effect of pyomelanin in larval settlement and metamorphosis of *M. coruscus*

**Figure 8:**
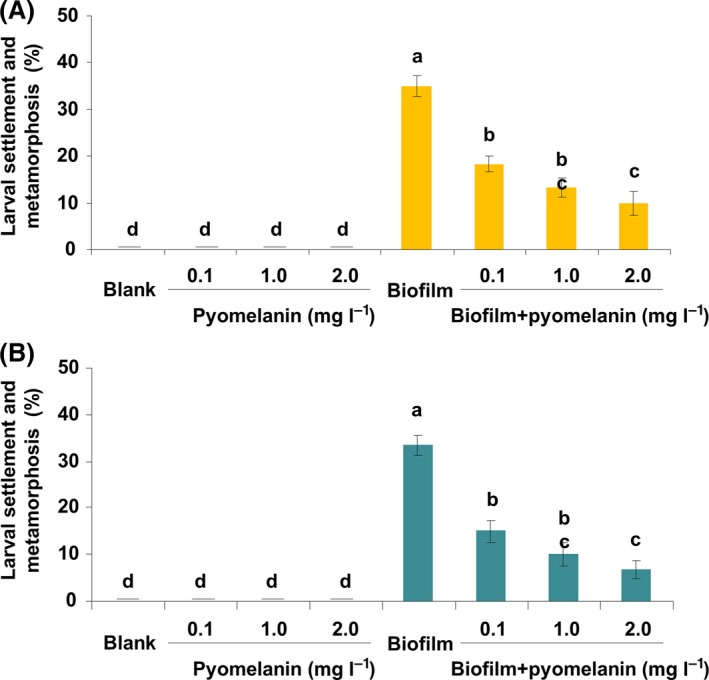
The inhibiting effect of pyomelanin in larval settlement and metamorphosis of *M. coruscus*. Larval settlement‐inducing activities of the pyomelanin extracted from the Δ*hmgA* mutant strain (A) and synthesized from homogentisic acid (B) with different concentrations in the absence or presence of bacterial biofilms. The lowercase letters indicate a significance level of *P* < 0.05.

To further confirm the inhibiting effect of pyomelanin in larval settlement and metamorphosis, we checked whether the biosynthesis pyomelanin extracted from the *P. lipolytica* Δ*hmgA* mutant and synthesized pyomelanin from the auto‐oxidation of HGA inhibit larval settlement and metamorphosis of *M. coruscus*. Our results showed that pyomelanin extracted from the *P. lipolytica* Δ*hmgA* mutant had a significant inhibiting effect on larval settlement and metamorphosis at all concentrations (Fig. 8A, Kruskal–Wallis test, *P* < 0.001). In the case of pyomelanin synthesized from the auto‐oxidation of HGA, significant inhibiting effect was also observed in all the concentrations tested (Fig. 8B, Kruskal–Wallis test, *P* < 0.001). No larval motility was observed when exposed the larvae to the extracted pyomelanin or *in vitro*‐synthesized pyomelanin at all the concentrations tested (data not shown). Taken together, these results demonstrate that the production of pyomelanin reduces the *M. coruscus* larval settlement and metamorphosis.

## Discussion

Pigments are organic compounds that confer characteristic colours to living tissues and are usually involved in vital processes (Liu and Nizet, 2009; Soliev *et al*., 2011). The *Pseudoalteromonas* genus has been divided into two clades based on differences in pigment production (Table S4). Pigmented species of this genus, such as *P. rubra*,* P. denitrificans*,* P. tunicata,* have been shown to exhibit strong antifouling activities (Egan *et al*., 2002; Holmstrom *et al*., 2002; Bowman, 2007). However, few evidences have been shown that the antifouling activities were directly induced by the pigment production. In this study, we first demonstrated that the pyomelanin overproduction mutant greatly reduced the *M. coruscus* settlement and metamorphosis. Furthermore, the purified pyomelanin from the Δ*hmgA* mutant which hyperproduces pyomelanin and the synthesized pyomelanin produced by the *in vitro* auto‐oxidation of HGA reduced the *M. coruscus* settlement and metamorphosis, suggesting an important ecological role of pyomelanin secreted by marine bacteria. Many marine bacteria are natural producers of melanin‐like compounds. Pyomelanin is one class of melanin derived from tyrosine via the intermediate HGA, and the formation of HGA is catalysed by 4‐HDDP; HGA is excreted, auto‐oxidized and polymerized to form pyomelanin. The present study demonstrated that *P. lipolytica* Δ*hmgA* mutant produces pyomelanin, similar to *Vibrio cholerae* and *Shewanella colwelliana* (Kotob *et al*., 1995; Valeru *et al*., 2009).

The physiological function of pyomelanin has been previously explored in the clinically important pathogens. For example, in *Legionella pneumophila*, the secreted pyomelanin conferred a ferric reductase activity which played an important role in iron uptake, thus enhancing growth under iron‐limiting conditions (Chatfield and Cianciotto, 2007; Zheng *et al*., 2013). In *Vibrio cholera*, pyomelanin production results in increased toxin‐co‐regulated pilus and cholera toxin expression and increases the ability of the bacteria to colonize the intestine of infant mice (Valeru *et al*., 2009). Although pyomelanin is related to the virulence and pathogenicity of pathogenic bacteria, pyomelanin production is also associated with numerous survival advantages. The primary advantage is thought to be protection against ultraviolet radiation. In *Burkholderia cenocepacia* and *Pseudomonas aeruginosa*, pyomelanin has been reported to scavenge free radicals and is thus able to protect cells from *in vitro* and *in vivo* sources of oxidative stress (Keith *et al*., 2007; Rodriguez‐Rojas *et al*., 2009). In *Bacillus anthracis*, the loss of *hmgA* can lead to the accumulation of pyomelanin, which protects *B. anthracis* cells from UV damage (Han *et al*., 2015). In *Shewanella oneidensis*, the release of pyomelanin into the surrounding environments could contribute to the survival in oxygen limited environment by increasing the anaerobic respiration capacity (Turick *et al*., 2009). Here we demonstrated that the secreted pyomelanin from marine bacteria also plays an important role in mediating the settlement and metamorphosis *of M. coruscus* and thus has its potential in control biofouling.

The *hmgA* variants that produce pyomelanin have been isolated from pathogenic bacteria during biofilm growth in a laboratory or from clinical isolates from infected patients (Boles *et al*., 2004; Keith *et al*., 2007; Valeru *et al*., 2009). For example, naturally occurring pyomelanin hyperproducing *Vibrio cholerae* variants have been found during growth under commonly used experimental culture conditions (Valeru *et al*., 2009; Wang *et al*., 2011). Variants of *Pseudomonas aeruginosa* that overproduced pyomelanin have been previously isolated from patients with cystic fibrosis and bronchiectasis (Drenkard and Ausubel, 2002), and they were also identified in biofilm communities grown in the laboratory (Boles *et al*., 2004; Rodriguez‐Rojas *et al*., 2009). The hyperproduction of pyomelanin in these variants is due to the loss or reduced activity of the key member of the l‐tyrosine catabolic pathway, HmgA. It has been reported that higher temperature could increase the mutation rate and contribute to bacteria evolution (Puurtinen *et al*., 2016). Moreover, variant subpopulations often emerge in bacteria during biofilm mode of growth (Stewart and Franklin, 2008). In this study, we found that the increased pyomelanin production under elevated temperature or during biofilm formation was neither due to a reduced expression of *hmgA* gene nor due to an increased expression of the *melA* gene. Thus, the increased pyomelanin production might be induced by the generation of the pyomelanin hyperproduction variants, such as *hmgA* mutant, in the biofilm of *P. lipolytica* under elevated temperature.

The *Pseudoalteromonas* genus is often found in association with eukaryotic hosts and adopted an attached lifestyle in the native marine environment (Holmstrom and Kjelleberg, 1999). Surface colonization of marine bacteria often involves biofilm formation and plays important role in numerous critical marine processes, including nutrient regeneration and organic matter remineralization (Dang and Lovell, 2016). Once forming biofilms, the establishment of concentration gradients of oxygen and nutrient could lead to the generation of genetic variants through mutation or recombination (Stewart and Franklin, 2008). In this study, *hmgA* variant of *P. lipolytica* was generated from laboratory‐cultured biofilms in nutrient‐rich SW‐LB medium; we hypothesized that similar mutations might also occur in the marine habitats where marine bacteria constantly face with the availability of the oxygen and nutrient. Indeed, variants isolated from laboratory‐cultured biofilms formed by pathogenic bacteria share similar mutations to those found in clinical isolates during infection (Drenkard and Ausubel, 2002; D'Argenio *et al*., 2007; Woo *et al*., 2012). *Pseudoalteromonas* strains that produce pigments were previously isolated in various marine environments. Of the 24 pigmented *Pseudoalteromonas* strains identified to date, five, namely *P. nigrifaciens* (Baumann *et al*., 1984), *P. aliena* (Ivanova *et al*., 2004), *P. distinct* (Ivanova *et al*., 2000), *P. carrageenovora* (Ivanova *et al*., 2000) and *P. haloplanktis* (Ivanova *et al*., 2000), have been characterized as melanin‐like pigment‐producing strains (Table S4). Enzymes responsible for pyomelanin production are generally found in *Pseudoalteromonas* strains according to the genome annotation in NCBI database (data not shown). Thus, it remains to be determined whether these melanin‐producing *Pseudoalteromonas* strains carry genetic variations in the *hmgA* gene or the production of the pyomelanin is induced at the transcriptional level.

Marine‐derived natural antifouling products are good potential antifoulants. The settlement of marine invertebrate larvae can be influenced by positive or negative cues produced by different marine organisms (Walters *et al*., 1996; Dobretsov *et al*., 2013). As previously reported, natural antifouling agents could be classified into six main groups according to their chemical structures: fatty acid‐related compounds, polyketide‐related compounds, terpenoid‐related compounds, steroid‐related compounds, alkaloid‐related compounds and non‐ribosomal polypeptide‐related compounds (Dobretsov *et al*., 2013; Qian *et al*., 2015). In addition, extracellular polymeric substances isolated from biofilms formed by *Pseudoalteromonas ulvae* and *Oceanobacillus iheyensis* have been shown to exhibit antifouling activities (Kavita *et al*., 2014; Brian‐Jaisson *et al*., 2016). In this study, we showed that pyomelanin exhibits strong antifouling activities. Pyomelanin is composed of complex polyphenolic heteropolymers and might represent a new group of chemical compounds that could serve as antifouling agents. The molecular mechanisms underlying the antifouling activity of pyomelanin as well as the physiological and ecological roles of pyomelanin production by marine *Pseudoalteromonas* in diverse marine ecosystems need to be further investigated.

## Experimental procedures

### Strains and growth conditions

*Escherichia coli* strain WM3064 strain was grown in Luria–Bertani (LB) medium containing 0.3 mM DAP (2,6‐diamino‐pimelic acid) at 37 °C (Dehio and Meyer, 1997). *Pseudoalteromonas lipolytica* SCSIO_04301 was isolated from sediment at 63 m deep in the South China Sea (Zeng *et al*., 2014). *P. lipolytica* Δ*hmgA* mutant strain used in this study has been constructed as we previously reported (Wang *et al*., 2015a,b). *P. lipolytica* was grown in 2216E medium (0.5% peptone, 0.1% yeast extract and different concentrations of the trace elements) (Becton, Dickinson and Company, Franklin Lakes, NJ, USA) or SW‐LB medium (1% tryptone and 0.5% yeast extract dissolved in sea water) at 25 °C unless indicated. Chloramphenicol (30 μg ml^−1^) was used for the maintenance of pBBR1MCS‐based plasmids (Zeng *et al*., 2015).

### Pigment production quantification

Wild‐type *P. lipolytica* strain was cultured to OD_600_ of 1.0 as planktonic stage or cultured by statically as biofilm formation for different days at different temperature. The cultures were centrifuged at the 17 000 *g* for 15 min after indicated days to remove cell pellet. The production of pigment in the supernatant was estimated by measuring the absorbance of the supernatant at 400 nm as previous reports have shown that pigment production can more easily be distinguished by measuring the absorbance at a lower wavelength (Ruzafa *et al*., 1995; Lagunas‐Munoz *et al*., 2006; Chatfield and Cianciotto, 2007). To monitor the secreted pigments, sea water, SW‐M9 (0.2% casamino acid dissolved in sea water) and SW‐LB medium supplemented l‐tyrosine or not were used to culture *P. lipolytica* by shaking or statically for different days, and the pigments were accessed by measuring the absorbance of the supernatant at 400 nm.

### Isolation of biofilm variants

For the isolation and quantification of biofilm variants, biofilms were harvested, uniformly homogenized and were plated onto SW‐LB agar plates as previously described (Zeng *et al*., 2015). A total of 5000 colonies were examined to screen for variants with altered colour morphology. Colour morphology of these variants was further examined after three rounds of overnight passaging.

### Whole‐genome resequencing

The P3 variant genome was sequenced by BGI Co., Ltd. (Shenzhen, Guangdong, China) using the whole‐genome shotgun method with the Illumina HiSeq 2000 sequencing platform as previously reported (Zeng *et al*., 2015). SNPs and InDels were detected based on an alignment of the assembly sequence and the wild‐type reference (Zeng *et al*., 2014).

### Complementation analysis

The broad‐host‐range vector pBBR1MCS was used to express the target genes in *P. lipolytica*. The coding region of the *hmgA* gene (GenBank accession number JDVB01000008.1) and its promoter region were amplified by PCR using the primer pair *hmgA*‐F (5′‐CGGAATTCTCGACCATATCGCTGCCATCACG‐3′) and *hmgA*‐R (5′‐CCCAAGCTTTTATACGTCCTGTTTACCTGTA‐3′) and ligated to pBBR1MCS‐Cm after enzymatic digestion with *EcoR*I and *Hind*III. The resulting recombinant plasmid was sequenced using the primers pBBR1MCS‐f and pBBR1MCS‐r and conjugated into *P. lipolytica* according to a previously described method (Wang *et al*., 2015a,b).

### FTIR analysis

The pigment of Δ*hmgA* mutant was extracted according to a previously published method with minor modifications (Schmaler‐Ripcke *et al*., 2009). Briefly, a 200 ml culture of Δ*hmgA* mutant was grown for 3 days in SW‐LB supplemented with 2.5 mM l‐tyrosine. Culture supernatant was collected by centrifugation and filter sterilization. The supernatant was acidified with 6 M HCl to a pH of 2.0 and allowed to precipitate overnight at room temperature. After centrifugation (16 500 *g*, 30 min), the pellet was resuspended in 2.5 ml of water at pH of 12 and dialysed in 3.5 kDa dialysis tubing for 24 hr against water. The dialysed pigment was then dried using the vacuum centrifugal concentrator (EYELA Tokyo Rikakikai Co Ltd CVE‐3110, Tokyo, Japan). Synthetic pyomelanin was used as a positive control compound in FTIR analysis. Synthetic pyomelanin was produced by auto‐oxidation of a 10 mM HGA (Tokyo Chemical Industry. Co. Ltd, Tokyo, Japan) at pH 10 with constant stirring for 3 days. Polymerization was stopped, and precipitation was started by adjusting the pH to 2 with 6 M HCl and further treated as described above. The pigment extracted from the Δ*hmgA* mutant and synthetic pyomelanin was used for FTIR analysis (Shimadzu Corporation, IR Affinity‐1, Kyoto, Japan).

### Culturing of the mussels

Plantigrades and pediveliger larvae of the mussel *Mytilus coruscus* were supplied by the Institute of Marine Science and Technology of Shengsi, Zhoushan, China. The plantigrades and pediveliger larvae of *M. coruscus* were cultured following a previously described method with some modifications (Wang *et al*., 2012; Yang *et al*., 2013, 2014a,b). Briefly, the plantigrades were transferred and cultured in aerated glass beakers with filtered sea water (acetate fibre filter, 1.2 μm pore size) at an initial density of 10 plantigrades ml^−1^ and fed a diet consisting of the algae *Isochrysis galbana* at a concentration of 10^5^ cells ml^−1^ day^−1^. Plantigrades with a shell length of 2.01 ± 0.04 mm and a shell height of 1.10 ± 0.03 mm were used for the settlement bioassays. Pediveliger larvae were transferred and cultured in aerated glass beakers with filtered sea water at an initial density of 5 larvae ml^−1^ and fed a diet consisting of the algae *I. galbana* at a concentration of 5 × 10^4^ cells ml^−1^ day^−1^. The plantigrades and pediveliger larvae were cultured for more than 1 week and then were used for the settlement bioassays.

### Mussel settlement bioassay

Biofilms of *P. lipolytica* and pigment production variants were prepared following a modified method (Zeng *et al*., 2015). Briefly, each strain was cultured in 2216E at 25 °C for 48 h and then cells were harvested. Cell pellet was washed three times by autoclaved filtered sea water (AFSW, GF/C filter, pore size = 1.2 μm; Whatman International Ltd., Maidstone, UK), and final cell density was adjusted to colony‐forming unit (CFU) of 10^6^ to 10^7^ per ml. Cell suspension was transferred to 24‐well plate and sterile glass Petri dishes with one piece of sterile glass slip. Bacteria that remained firmly attached on surfaces of plates and the glass slips were viewed as irreversible attached bacterial biofilms. For each strain, six replicates were conducted. For the plantigrade settlement bioassay, ten plantigrades were transferred into individual glass Petri dishes containing 20 ml of AFSW and a glass slip with monospecific bacterial biofilm (Yang *et al*., 2014a,b). Six replicates were conducted. The inducing activity was determined by the percentage of settled plantigrades after 12 h. The settled plantigrades were verified after attachment to the surface with byssal threads. A glass slip without biofilm was included as a negative control in all assays. For the pediveliger larval settlement bioassay, ten pediveliger larvae were added into each 24‐well plate containing bacterial biofilms and 1.5 ml of pyomelanin solution of different concentrations (Yang *et al*., 2011, 2014a,b). A group of pediveliger larvae were exposed to bacterial biofilms as a positive control. Six replicates were conducted. In addition, pediveliger larvae were also exposed to pyomelanin from biosynthesis and chemical synthesis without the addition of bacterial biofilms as a control. The inducing activity was evaluated by the percentage of metamorphosed individuals (post‐larvae) after 72 h following our previously developed method (Wang *et al*., 2012). Post‐larvae were examined at 100× magnification under an Olympus stereoscopic microscope and the images of the pediveliger larvae and post‐larvae of *M. coruscus* are shown in Fig. S3. A negative control was included with twenty pediveliger larvae and 1.5 ml of AFSW in the absence of attached biofilm. Assays were conducted at 18 °C in darkness with six replicates for each condition.

### Quantitative real‐time reverse‐transcription PCR

Cells were collected and total RNAs were isolated as described previously (Ren *et al*., 2004). The quantitative real‐time reverse‐transcription PCR (qRT‐PCR) reaction was performed using the Super‐Script III Platinum SYBR Green One‐Step qRT‐PCR Kit (Thermo Fisher Scientific, Waltham, MA, USA). The level of *rrsE* transcript was used to normalize the gene expression data. Primer pairs for qRT‐PCR include (5′‐TGATAAACCGGAGGAAGGTG‐3′) and (5′‐TTCATGGAGTCGAGTTGCAG‐3′) for *rrsE* (5′‐GCGCAGATAATCGCAGAACA‐3′) and (5′‐TGCCGCCATTGTGACTAGAC‐3′) for *hmgA* and (5′‐CCGGGTGTACAACACTTAGCATT‐3′) and (5′‐CTGGCTATCGACAAGAATTTGGT‐3′) for *melA*.

## Conflict of interest

None declared.

## Supporting information


**Fig. S1.** The visible light spectra of the pigments collected from the superanatnat of the *P. lipolytica* Δ*hmgA* mutant grown for 2 days and wild‐type *P. lipolytica* cultured statically in SW‐LB medium at 25 °C for 10 days.
**Fig. S2.** *P. lipolytica* Δ*hmgA* mutant strain produced pyomelanin.
**Fig. S3.** The settlement and metamorphosis of the pediveliger larvae of *Mytilus coruscus*.
**Table S1.** Point mutations revealed by whole‐genome re‐sequencing of the pyomelanin hyper‐production variant P3 isolated from *P. lipolytica* biofilms.
**Table S2.** Tyrosine metabolic pathway in *P. lipolytica* predicted by compared with *Pseudomonas putida* NBRC 14164.
**Table S3.** Identification of four l‐tyrosine catabolic pathway related enzymes in the genome of the represented *Pseudoalteromonas* strains.
**Table S4.** List of pigmented *Pseudoalteromonas* strains isolated from various marine or other habitats.
**Table S5.** The fold change of the *hmgA* and *melA* transcription level in *P. lipolytica* under stress conditions compared to planktonic stage at 25 °C.
