# Supplementary material for: Pyomelanin from Pseudoalteromonas lipolytica reduces biofouling
Source: Microb Biotechnol. 2017 Aug 22;10(6):1718–31. doi: 10.1111/1751-7915.12773 (PMC5658579; doi:10.1111/1751-7915.12773)
Supplement: Supplementary file 1 — Fig. S1. The visible light spectra of the pigments collected from the superanatnat of the P. lipolytica ΔhmgA mutant grown for 2 days and wild‐type P. lipolytica cultured statically in SW‐LB medium at 25 °C for 10 days. Fig. S2. P. lipolytica ΔhmgA mutant strain produced pyomelanin. Fig. S3. The settlement and metamorphosis of the pediveliger larvae of Mytilus coruscus. Table S1. Point mutations revealed by whole‐genome re‐sequencing of the pyomelanin hyper‐production variant P3 isolated from P. lipolytica biofilms. Table S2. Tyrosine metabolic pathway in P. lipolytica predicted by compared with Pseudomonas putida NBRC 14164. Table S3. Identification of four l‐tyrosine catabolic pathway related enzymes in the genome of the represented Pseudoalteromonas strains. Table S4. List of pigmented Pseudoalteromonas strains isolated from various marine or other habitats. Table S5. The fold change of the hmgA and melA transcription level in P. lipolytica under stress conditions compared to planktonic stage at 25 °C. [file MBT2-10-1718-s001.docx]

**SUPPLEMENTARY FILE**

**Pyomelanin from *Pseudoalteromonas lipolytica* Reduces Biofouling**

Zhenshun Zeng^1†^, Xing-Pan Guo^2†^, Xingsheng Cai^1^, Pengxia Wang^1^, Baiyuan Li^1^,

Jin-Long Yang^2*^, Xiaoxue Wang^1*^

^1^Key Laboratory of Tropical Marine Bio-resources and Ecology, Guangdong Key Laboratory of Marine Materia Medica, RNAM Center for Marine Microbiology, South China Sea Institute of Oceanology, Chinese Academy of Sciences, Guangzhou, China

^2^Key Laboratory of Exploration and Utilization of Aquatic Genetic Resources, International Research Center for Marine Biosciences, Shanghai Ocean University, Shanghai, China

^†^These authors contributed equally to this work

*To whom correspondence should be addressed. E-mail: [xxwang@scsio.ac.cn](mailto:xxwang@scsio.ac.cn); [jlyang@shou.edu.cn](mailto:jlyang@shou.edu.cn); Tel. +86 20 89267515; Fax +86 20 89235490.

**Table S1.** Point mutations revealed by whole-genome re-sequencing of the pyomelanin hyper-production variant P3 isolated from *P. lipolytica* biofilms.

| Mutation number | Annotation number | Gene name | Gene products | Type of mutation | Wild-type | P3 variant | Protein position in mutated gene |
| --- | --- | --- | --- | --- | --- | --- | --- |
| M1 | AT00_01635 | *gcvP* | glycine dehydrogenase | nonsyn | C | A | 711 |
| M2 | AT00_01945 | *phrB* | FAD-binding protein | nonsyn | G | T | 245 |
| M3 | intergenic |  |  |  | G | T |  |
| M4 | AT00_07185 |  |  | nonsyn | G | A | 35 |
| M5 | AT00_08765 |  | histidine kinase | nonsense | T | A | 236 |
| M6 |  | *thiDE* |  | nonsyn | T | A | 436 |
| M7 | AT00_12500 | *cirA* | tonB-dependent  receptor | nonsyn | G | T | 683 |
| M8 | AT00_12885 | *acrB* | multidrug transporter | nonsyn | C | G | 680 |
| M9 | intergenic |  |  |  | A | T |  |
| M10 | AT00_15270 | *sgaA* | glyoxalase | syn | G | T | 100 |
| M11 | AT00_15690 | *hmgA* | homogentisate 1,2-dioxygenase | nonsyn | G | A | 197 |
| M12 | AT00_16625 |  |  | nonsyn | A | T | 902 |

**Table S2**. Tyrosine metabolic pathway in *P. lipolytica* predicted by compared with *Pseudomonas putida* NBRC 14164.

| Gennbank ID | Gene name | Gene function | Homolog | Coverage | Identity |
| --- | --- | --- | --- | --- | --- |
| PP4_07400 | *hmgA* | homogentisate-1,2-dioxygenase | AT00_15690 | 97% | 57% |
| PP4_07410 | *fahA* | fumarylacetoacetate hydrolase | AT00_02285 | 99% | 53% |
| PP4_07420 | *maiA* | maleylacetoacetate isomerase | AT00_15695 | 98% | 43% |
| PP4_28470 | *melA* | 4-hydroxyphenylpyruvate dioxygenase | AT00_02290 | 97% | 45% |
| PP4_13300 | *phhA* | phenylalanine hydroxylase | AT00_08070 | 98% | 62% |
| PP4_22370 | *tat* | tyrosine aminotransferase | AT00_08610 | 100% | 41% |

**Table S3.** Identification of four L-tyrosine catabolic pathway related enzymes in the genome of the represented *Pseudoalteromonas* strains.

| ***Pseudoalteromonas* strain** | Chromosome I | |  | Chromosome II | |
| --- | --- | --- | --- | --- | --- |
|  | MelA | FahA |  | HmgA | MaiA |
| ***P. lipolytica* SCSIO 04301** | AT00_02290 | AT00_02285 |  | AT00_15690 | AT00_15695 |
| ***P. haloplanktis* TAC125** | PSHAa2168 | PSHAa2169 |  | PSHAb0338 | PSHAb0337 |
| ***P. rubra* ATCC 29570 ^T^** | PRUB_19507 | PRUB_19512 |  | PRUB_23396 | PRUB_23391 |
| ***P. tunicate* D2^T^** | PTD2_11669 | PTD2_11674 |  | PTD2_03416 | PTD2_03421 |
| ***P. flavipulchra* JG1** | PflaJ_010100008480 | PflaJ_010100008475 |  | PflaJ_010100004606 | PflaJ_010100004601 |
| ***P.undina* NCIMB 2128 ^T^** | PUND_05219 | PUND_05214 |  | PUND_09464 | PUND_09459 |
| ***P.* sp SCSIO11900** | BG00_04605 | BG00_04610 |  | BG00_16885 | BG00_16880 |
| ***P.* sp SM9913** | PSM_0972 | PSM_0971 |  | PSM_0404 | PSM_0403 |

**Table S4.** List of pigmented *Pseudoalteromonas* strains isolated from various marine or other habitats.

| **Taxon** | **Strain** | **Pigment** | **Growth** | **Source** |
| --- | --- | --- | --- | --- |
| ***P. aliena*** | **SW 19^T^** | **Melanin-like** | **4 to 29 °C** | **Seawater of Amursky Bay, Sea of Japan** |
| ***P. distincta*** | **KMM 638^T^** | **Melanin-like** | **30 °C** | **Marine sponge collected at a depth of 350m, Komandorskie Islands, Russia** |
| ***P. carrageenovora*** | **ATCC 43555^T^** | **Melanin-like** | **5~35 °C** | **Seawater, Nova Scotia, Canada** |
| ***P. haloplanktis*** | **IAM 12915 ^T^** | **Melanin-like** | **4 to 35 °C** | **Sessile form found associated with marine phytoplankton, Russia** |
| ***P. nigrifaciens*** | **ATCC 19375^T^** | **Melanin-like** | **4 to 30 °C** | **Saline butter, Sea of Japan, Russia** |
| *P. phenolica* | O-BC 30^T^ | Brown | 18 to 37 °C | Sea water, Ogasawara Island, Japan |
| *P. aurantia* | ATCC 33046^T^ | Orange | 4 to 30 °C | Surface seawater off Nice, France |
| *P. citrea* | ATCC 29719^T^ | Yellow | 10 to 30 °C | Surface seawater in the Mediterranean Sea |
| *P. denitrijicans* | ATCC 43337^T^ | Blue, purple or red color | 4 to 22 °C | Water samples at depths of 90 to 100m, fjord system, Norway |
| *P. luteoviolacea* | ATCC 33492^T^ | Violet | 10 to 30 °C | Surface seawater in the Mediterranean Sea |
| *P. rubra* | ATCC 29570^T^ | Red or pink | 10 to 37 °C | Mediterranean Sea near Nice |
| *P. piscicida* | ATCC 15057^T^ | Yellow or orange | 20 to 40 °C | Red-tide seawater, Indian river at Melbourne, Florida |
| *P. arctica* | A 37-1-2^T^ | Slightly orange | 4 to 25 °C | Seawater samples, Spitzbergen in the Arcitc, Norway |
| *P. byunsanensis* | FR 1199^T^ | Violet | 10 to 40 °C | Tidal flat sediment of Byunsan, Republic of Korea |
| *P. flavipulchra* | KMM 3630^T^ | Orange | 10 to 44 °C | Surface seawater off Nice, France |
| *P. maricaloris* | KMM 636^T^ | Lemon yellow | 10 to 37 °C | Australian sponge, Coral Sea, Pacific Ocean |
| *P. paragorgicola* | KMM 3548^T^ | Pale orange | 4 to 30 °C | Pacific Ocean at a depth of 202m |
| *P. peptidolytica* | F12-50-A1^T^ | Yellow | 15 to 40 °C | Surface seawater, Sea of Japan |
| *P. ruthenica* | KMM 300^T^ | Pale orange | 10 to 35 °C | Mussel and scallop, Sea of Japan |
| *P. spongiae* | UST010723-006^T^ | Pale orange | 12 to 44 °C | Surface of the sponge *Mycale adhaerens* in Hong Kong waters |
| *P. ulvae* | UL 12^T^ | Dark purple | 23 °C | Marine alga *U.lactuca*, rocky intertidal zone, Australia |
| *P. atlantica* | ATCC 19262^T^ | Pale yellow orange | 5~35 °C | Seaweed, *Rhodomeniapalmate*, Canada |
| *P. tunicata* | D2^T^ | Dark-green | 28 °C | Adult tunicate *Cionaintestinalis*, western coast of Sweden |
| *P. xiamenensis* | Y2^T^ | Dark red | 10 to 40 °C | Surface seawater, Yundang Lake, Xiamen, China |

Note:

1. *P. aurantia* ATCC 33046^T^ formed a brownish orange center and a greenish edge in MA medium.
2. *P. ulvae* UL12^T^ formed a dark purple colonies in VNSS medium while a white one in LB20 or TSB with 2% NaCl.
3. Growth of *P. tunicata* D2^T^ on VNSS medium results in dark-green pigmented, while white in TSB medium.

**Table S5.** The fold change of the *hmgA* and *melA* transcription level in *P. lipolytica* under stress conditions compared to planktonic stage at 25°C.

| **Gene analyzed** | **Conditions** | **C_T_** | **ΔCT** | **Fold-change** |
| --- | --- | --- | --- | --- |
| *rrsE*  (house-keeping) | planktonic 25°C (OD_600_=1.0) | 12.99 ± 0.11 |  |  |
|  | planktonic 37°C (30 min) | 12.70 ± 0.44 |  |  |
|  | Biofilm 25°C (3 day) | 12.95 ± 0.14 |  |  |
| *hmgA* | planktonic 25°C (OD_600_=1.0) | 27.12 ± 0.84 | 14.13 ± 0.85 | 1 |
|  | planktonic 37°C (30 min) | 25.80 ± 0.51 | 13.09 ± 0.67 | 2.0 ± 0.29 |
|  | Biofilm 25°C (3 day) | 27.26 ± 0.93 | 14.31 ± 0.94 | -1.14 ± 0.85 |
| *melA* | planktonic 25°C (OD_600_=1.0) | 23.51 ± 0.59 | 10.51 ± 0.6 | 1 |
|  | planktonic 37°C (30 min) | 23.93 ± 0.70 | 11.23 ± 0.83 | -1.64 ± 0.9 |
|  | Biofilm 25°C (3 day) | 25.79 ± 0.85 | 12.84 ± 0.86 | -5.01 ± 2.8 |

**Fig S1.** The visible light spectra of the pigments collected from the superanatnat of the *P. lipolytica* Δ*hmgA* mutant grown for 2 days and wild-type *P. lipolytica* cultured statically in SW-LB medium at 25 °C for 10 days. Data are from three independent cultures and One s.d. is shown.

**
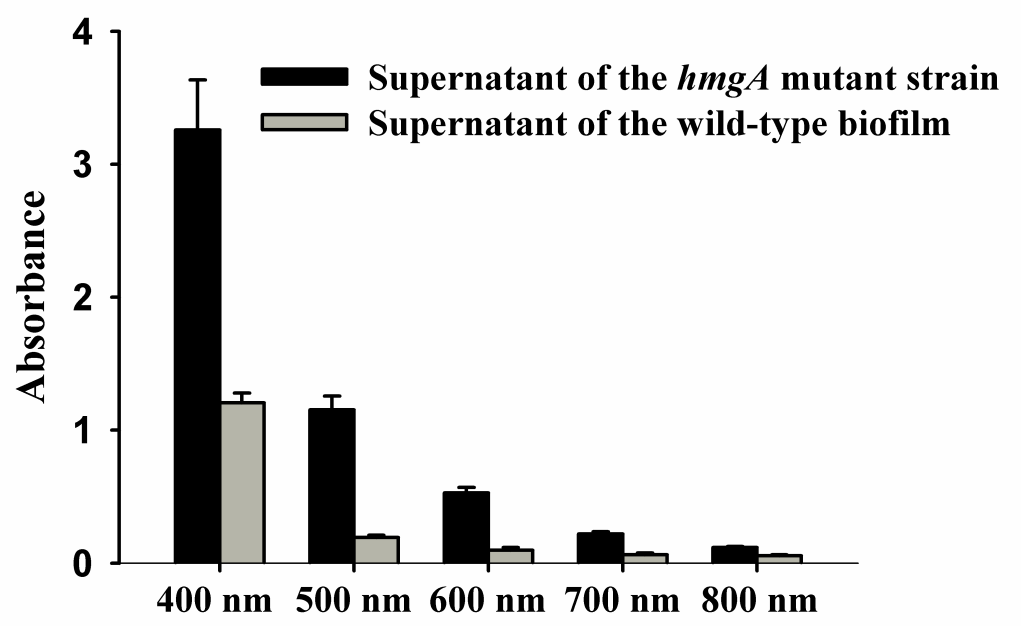
**

**Fig S2.** *P. lipolytica* Δ*hmgA* mutant strain produced pyomelanin. FITR spectrum overlay from synthetic melanin and dried extracellular pigment extracted from Δ*hmgA* mutant of *P. lipolytica* SCSIO 04301.

**
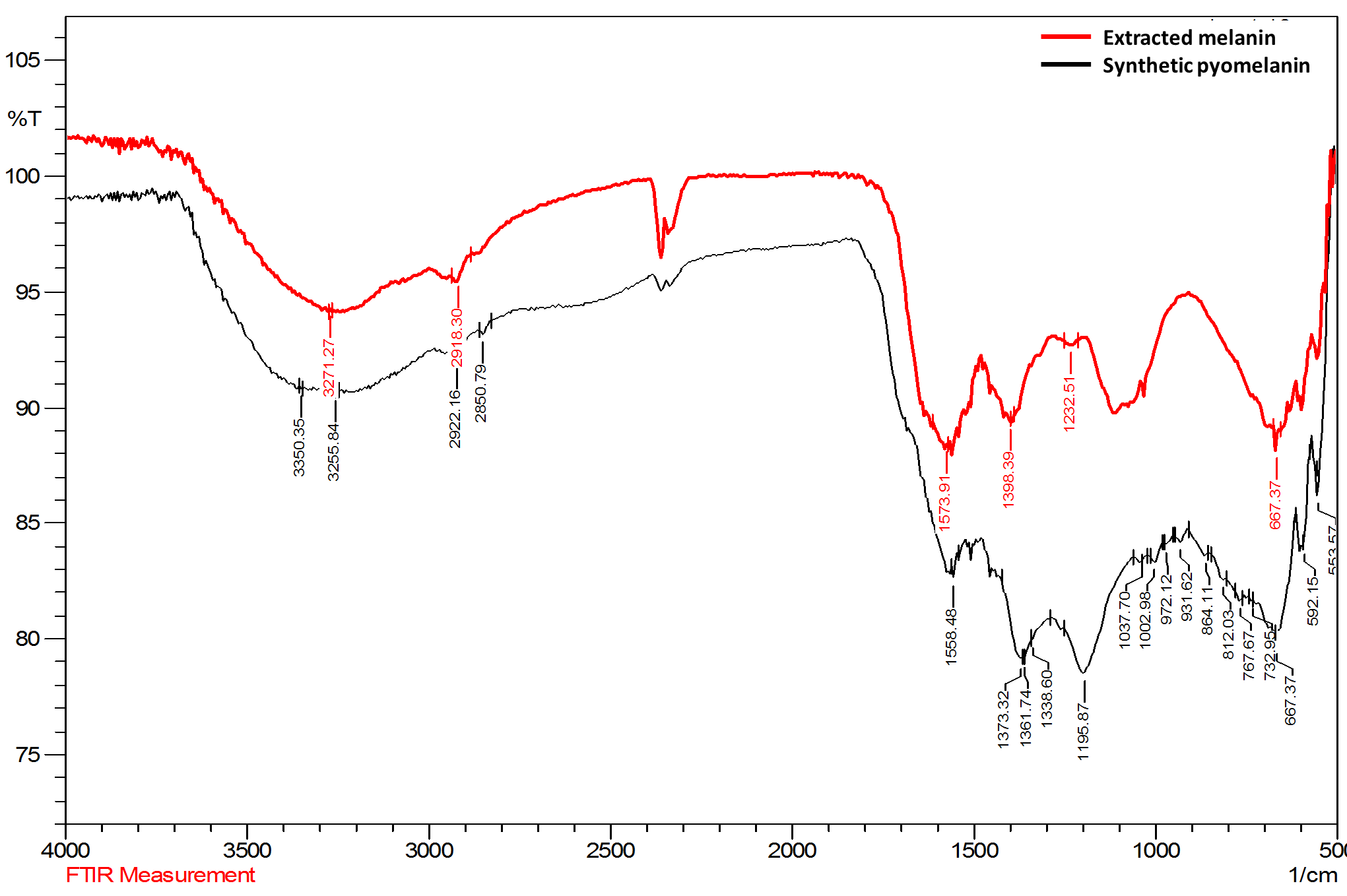
**

**Fig S3.** The settlement and metamorphosis of the pediveliger larvae of *Mytilus coruscus*.

**
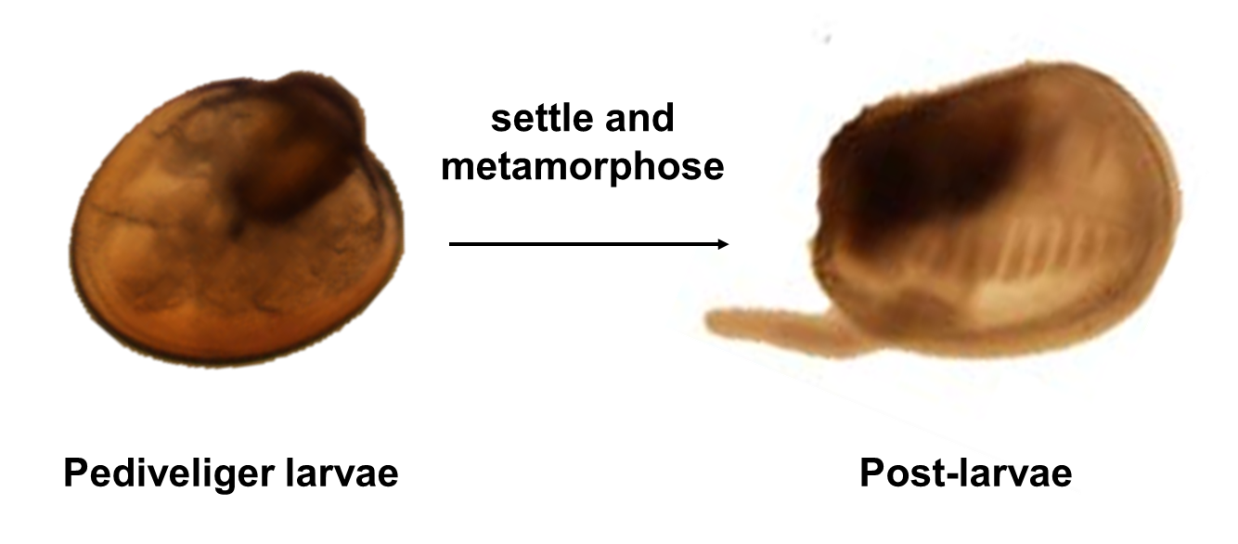
**
